# Safety of Mesenchymal Stem Cells for Clinical Application

**DOI:** 10.1155/2012/652034

**Published:** 2012-05-16

**Authors:** Youwei Wang, Zhi-bo Han, Yong-ping Song, Zhong Chao Han

**Affiliations:** ^1^State Key Laboratory of Experimental Hematology, Institute of Hematology and Hospital of Blood Diseases, Chinese Academy of Medical Sciences and Peking Union of Medical College, Tianjin 300020, China; ^2^Department of Hematology, Affiliated Tumor Hospital of Zhengzhou University, Zhengzhou 450052, China

## Abstract

Mesenchymal stem cells (MSCs) hold great promise as therapeutic agents in regenerative medicine and autoimmune diseases, based on their differentiation abilities and immunosuppressive properties. However, the therapeutic applications raise a series of questions about the safety of culture-expanded MSCs for human use. This paper summarized recent findings about safety issues of MSCs, in particular their genetic stability in long-term *in vitro* expansion, their cryopreservation, banking, and the role of serum in the preparation of MSCs.

## 1. Introduction

It has been shown that the transplantation of mesenchymal stem cells (MSCs) could be an effective therapy for many diseases including blood disease, acute respiratory distress syndrome, spinal cord injury, liver injury, and critical limb ischemia [1–4]. To date, hundreds of clinical trials using MSCs have been registered in the database (http://www.clinicaltrials.gov/) of the US national institutes of health. Furthermore, a number of nonregistered clinical studies using MSCs are being performed in many countries. 

The general practice includes the isolation of MSCs from various tissues (including bone marrow, adipose tissue, placenta umbilical cord, umbilical cord blood, peripheral blood, and dental pulp) and the cell expansion under *in vitro* culture conditions. The complications in the utilization of MSCs as therapeutic tools* in vivo* arose due to the experimental artifacts introduced by inconsistent cell culture protocols. Actually, most MSCs used for clinical trials are prepared in research laboratories, lacking sufficient preclinical studies and manufacturing quality control. Moreover, laboratories around the world lack an internationally standardized practice for *in vitro* expansion of MSCs, resulting in heterogeneous populations of cells and inconsistent results, both in experimental studies and clinical trials.

In addition, although MSCs have been used in both autologous and allogeneic settings, most clinical applications of MSCs are in fact personalized therapies in which the patient receives administration of MSCs provided by different donor and/or different preparation. This necessitates the establishment of standardized manufacture guidelines for the isolation, expansion, preservation, and delivery of MSCs that display minimal variability in their production and assumes large-scale produced MSCs as “cell medicine” for safety evaluation and clinical applications.

## 2. Expansion and Genetic Stability of MSCs

Primary MSCs are rare in human tissues. The frequency of MSCs is approximately 1/10^6^ nucleated cells in adult bone marrow and 1/10^4^ nucleated cells in umbilical cord [5]. The number of MSCs has been noted to decrease with age. When grouped by decade, a significant decrease in MSCs per nucleated bone marrow cell could be observed, with 10-fold decrease from birth to teens and another 10-fold decrease from teens to elderly [6].

Despite limited number, MSCs can be expanded to a high level in long-term culture system, which permits a large-scale production of MSCs for clinical application. Usually, the adult bone marrow MSCs (BMMSCs) can grow identically in culture for 6–10 passages, whereas placenta umbilical cord MSCs can undergo 30 to 40 passages. It is known that prolonged culture of human embryonic stem cells (ESCs) can lead to adaptation and acquisition of chromosomal abnormalities [7–12]. The induced pluripotent stem cells (iPSCs) undergo deletions of tumor-suppressor genes during the process of reprogramming, while duplications of oncogenic genes aroused in culture [13]. It remains unclear whether the “culture-adapted” MSCs undergo adaptive transformation during long-term passaging *in vitro*. 

Previous data obtained from high-resolution analysis show that the *in vitro* expanded human BMMSCs are devoid of DNA copy number aberrations [14, 15]. However, senescence-associated modification at specific CpG sites has been observed in MSC during culture expansion. The key evidence for transformation based on DNA fingerprinting has not been presented in the studies in which the authors claimed that MSCs underwent malignant transformation in cultivation [16–25]. More data are therefore needed to evaluate the genomic stability of MSCs during prolonged culture *in vitro*. The highresolution of genetic analysis including balanced and unbalanced genetic change is an important method to determine the possibility of transformation of MSCs. If CNV or SNP change is detected after long-term cultivation, it refers not only to the occurrence of mutation but also to the mutation providing a survival (senescence or apoptosis resistance) or growth advantage more or less. Even if there is no difference between early- and late-passage MSCs in genome, it does not imply the absence of genomic alteration during long-term cultivation. The mutated MSCs without survival or growth advantage will be diluted in the process of cultivation and become undetectable after long-term cultivation. By contrast, the mutated MSCs with growth advantage or senescence resistance are of more risk for clinical application.

## 3. Tumor Formation

Stem cells posses some features of cancer cells including long lifespan, relative apoptosis resistance, and ability to replicate for extended periods of time. In addition, similar growth regulators and control mechanisms are involved in both cancer and stem cell maintenance. Therefore, stem cells may undergo malignant transformation which is often seen as a key obstacle to the safe use of stem-cell-based medicinal products.

Some previous studies have described spontaneous transformation of MSCs *in vitro*. However, almost all of them have not provided solid evaluation of the same origin of normal MSCs and their transformed counterparts. Actually, most of the spontaneous malignant transformed MSCs are cross-contained by HT1080, HEla, or other tumor cell lines [16, 17, 20, 21, 23–25]. There is no enough evidence for tumorigenicity of MSCs expanded *in vitro*. 

To address the safety issues, we conducted several GLP-compliant *in vivo* toxicity studies using NOD mice, NOD/SCID mice, guinea pigs, rabbits, and monkey models. UC-MSCs from master MSCs bank (passage 2, P2) were thawed and cultured for additional five passages (P7) and eleven passages (P13). At the end of P7 or P13, an approximate number of 6 × 10^9^ or 5 × 10^12^ UC-MSCs were, respectively, harvested, allotted, and cryopreserved until use. For tumorigenic study, UC-MSCs at a dose of 1 × 10^7^/mouse were subcutaneously transplanted into both NOD mice and NOD/SCID mice. No tumor formation was observed two months after cell transplantation in these animals. The effect of transplanted UC-MSCs on tumor growth was then studied using the Nod mice which were previously injected with K562 cells to induce leukemic tumors. Two injections (two-week interval) of different doses of UC-MSCs resulted in a significant inhibition of K562 tumor growth in the mice bearing leukemic tumors. These *in vivo* results are consistent with our *in vitro* results showing a potent inhibitory effect of UC-MSCs on the proliferation of K562 and HL-60 cells without inducing apoptosis [26].

In an effort to evaluate the overall toxicology of UC-MSCs, we have performed an *in vivo* study in cynomolgus monkeys receiving repeated administrations of UC-MSC. The administration of UC-MSC was done by intravenous injection once every two weeks for six weeks, with a dose of 2 × 10^6^ or 1 × 10^7^ cells/kg body weight. All animals survived until scheduled euthanasia. No significant MSCs-related changes were found in body weights, clinical signs, hematological/biochemical values, organ weights, or histopathological findings. The results of this toxicity study indicated that the transplantation of UC-MSC did not affect the general health of cynomolgus monkeys [27].

Moreover, the vast majority of clinical trials conducted with MSCs in regenerative medicine applications have not reported major health concerns. Centeno et al. report that two groups of patients (group 1: *n* = 50; group 2: *n* = 290) between 2006 and 2010 were treated for various orthopedic conditions with culture-expanded, autologous BMMSCs. Cells were cultured in monolayer culture flasks using an autologous platelet lysate technique and reinjected into peripheral joints or into intervertebral discs with use of c-arm fluoroscopy. Using both intensive high-field MRI tracking and complications surveillance in 339 patients, no neoplastic complications were detected at any stem cell reimplantation site [28]. MSCs-based therapy has also already been used in other human disease settings, such as graft-versus-host and cardiac disease, with initial reports indicating a good safety profile. These findings indicate the lack of solid evidence for malignant transformation *in vivo* following implantation of MSCs for clinical use. Further studies will be required to determine if MSCs can help tumor formation and related mechanisms. Although MSCs with chromosomal alterations did not show any sign of malignant transformation either *in vitro* or *in vivo* [29], it remains uncertain that acquired mutations will induce cellular transformation during the prolonged culture. There exists the possibility that MSCs gain copy number variation during prolonged expansion. Thus, it is necessary to conduct aCGH or SNP array to evaluate the genomic integrity of MSCs before clinical application.

## 4. Cryopreservation and Banking of MSCs

To isolate and produce in large scale the MSCs for clinical use, standardized preparation processes and long-term storage of MSCs isolated from different sources are needed for future clinical applications [30–32].

Since HLA-matched adult organ donors are not always available, stem cells derived from several birth-associated perinatal tissues—including cord blood, placenta, and umbilical cord—can be banked as a safeguard against future life-threatening conditions. The clinical grade production and preservation of perinatal MSCs necessitates adhering to cGMP (current Good Manufacture Practices) to insure the delivery of a “cell drug” that is not only safe but also reproducible and efficient. Cryopreservation of cells permits the transportation of cells between sites, as well as completion of safety and quality control testing. Taken together, banking of perinatal MSCs includes the donor determination and sample collection, primary cell isolation and microbiological testing, the master stem cell selection, cell expansion, cryopreservation and banking, and the large-scale cell expansion and preparation of final stem cell products. Strict standards and management are vital to make stem cell bank work well. Validated SOPs (Standard Operation Procedures) with quality assurance programs are the key factor of a well-designed bank of MSCs. Maintenance of viability, biological characteristics, and sterility makes the banked MSCs safety and “ready to use.”

It has been demonstrated that cryopreservation does not change the biological behavior of MSCs such as differentiation, growth, and surface marker [33]. Serum and dimethyl sulfoxide (DMSO) are used in research laboratory as cryoprotectant. The major challenge of freezing MSCs is the toxicity of cryoprotectant in clinical use. The toxicity of DMSO is overestimated as it could be weakened by diluting cyropreserved MSCs before clinical use. FBS (fetal bovine serum), BSA (bovine serum albumin), or HSA (human serum albumin) should not be an alternative cyroprotectant for DMSO because of a risk of contamination with human or animal viruses.

## 5. Serum-Containing and Serum-Free Cultivations

*In vitro* expansion of MSCs is conventionally achieved in medium containing FBS and is increased by addition of growth factors. However, for widespread clinical applications, contact of MSCs with serum must be minimized since it is a putative source of prion or virus transmission. Serum is the most uncertain factor in the expansion of clinical grade MSCs. Considering the batch-to-batch variability and possibility of viral contamination, some replacement of FBS such as human AB serum or platelet lysates cannot be considered as better choice for producing MSCs for clinical use [34]. Chemical-defined, xeno-free, serum-free medium (SFM) may conquer all the problems of FBS. Furthermore, comparing serum-contained medium, some evidence supported that SFM provided an adjuvant for maintenance of chromosomal stability in BMMSCs and adipose-derived MSCs [35]. Another study focus on mouse embryo fibroblasts showed that predominantly diploid karyotype was maintained in serum-free culture even at PD60. Aneuploid karyotype was induced by the addition of serum [36]. Probably the uncontrolled mitogenic stimulation in serum can lead to strong genetic instability. However, expansion of MSCs in SFM remains an unsolved question. SFM has its own shortcoming in the preparation of clinical grade MSCs. The attachment of MSCs in SFM needs the coating of fibronectin or other substrates which contained components of human origin and batch-to-batch variability and cannot be well defined chemically.

In fact, SFM is not as well as that some commercial companies claimed. Based on our own data, UC-MSCs proliferate more slowly in SFM than in FBS-contained media. Sometimes, UC-MSCs cannot be expanded in SFM. A number of studies have revealed that MSCs can be expanded in FBS contained medium without transformation [14]. If MSCs are expanded in SFM, the similar safety studies are needed to determine if the serum-free system affects the genetic stability of MSCs and cause tumor formation.

In summary, the safety remains one of the main concerns in cell therapy. The production of safe cell products requires an entire process supervising to make sure the cells maintain overall phenotype, functional potential, and to ensure the cultured cells remain untransformed and no microbiological contaminations. Therefore, MSCs banking and cell products manufacturing and corresponding quality control system procedures must be applied for assuring the safety and efficiency of the final cell products. Moreover, we cannot only rely on biologists to produce MSCs which fulfils all of the requirements for clinical application. Cell engineering technologies are needed in the translation from expansion of MSCs in laboratory to large-scale manufacture in cell factory.
